# A defined diet for pre-adult Drosophila melanogaster

**DOI:** 10.1038/s41598-024-57681-z

**Published:** 2024-03-23

**Authors:** Felipe Martelli, Annelise Quig, Sarah Mele, Jiayi Lin, Tahlia L. Fulton, Mia Wansbrough, Christopher K. Barlow, Ralf B. Schittenhelm, Travis K. Johnson, Matthew D. W. Piper

**Affiliations:** 1 School of Biological Sciences, Monash University, Clayton, VIC 3800 Australia; 2 Monash Proteomics and Metabolomics Platform, Monash Biomedicine Discovery Institute & Department of Biochemistry and Molecular Biology, Monash University, Clayton, VIC 3800 Australia; 3 Department of Biochemistry and Chemistry and La Trobe Institute for Molecular Science, La Trobe University, Bundoora, VIC 3086 Australia; 4 Present Address: School of BioSciences, The University of Melbourne, Melbourne, VIC 3052 Australia

**Keywords:** Holidic diet, Lipids, Amino acids, Acetate, Fruit flies, Developmental timing, Precision nutrition, Nutrigenomics, Genetics, Development, Drosophila, Disease model, Metabolism, Metabolic diseases

## Abstract

*Drosophila* melanogaster is unique among animal models because it has a fully defined synthetic diet available to study nutrient-gene interactions. However, use of this diet is limited to adult studies due to impaired larval development and survival. Here, we provide an adjusted formula that reduces the developmental period, restores fat levels, enhances body mass, and fully rescues survivorship without compromise to adult lifespan. To demonstrate an application of this formula, we explored pre-adult diet compositions of therapeutic potential in a model of an inherited metabolic disorder affecting the metabolism of branched-chain amino acids. We reveal rapid, specific, and predictable nutrient effects on the disease state consistent with observations from mouse and patient studies. Together, our diet provides a powerful means with which to examine the interplay between diet and metabolism across all life stages in an animal model.

## Introduction

Nutrition is a major environmental factor that affects animal physiology, metabolism, and influences traits such as survival and fitness^1^. Understanding how each nutrient contributes to the animal phenotype remains a major challenge in biology due to the complex composition of food. Animal models such as the vinegar fly *Drosophila melanogaster* permit wide-scale testing of genotype-environment interactions and their molecular basis. These attributes are key in medical and biological sciences due to the shared physiological and metabolic processes in flies and humans^2–5^.

**Figure 1:**
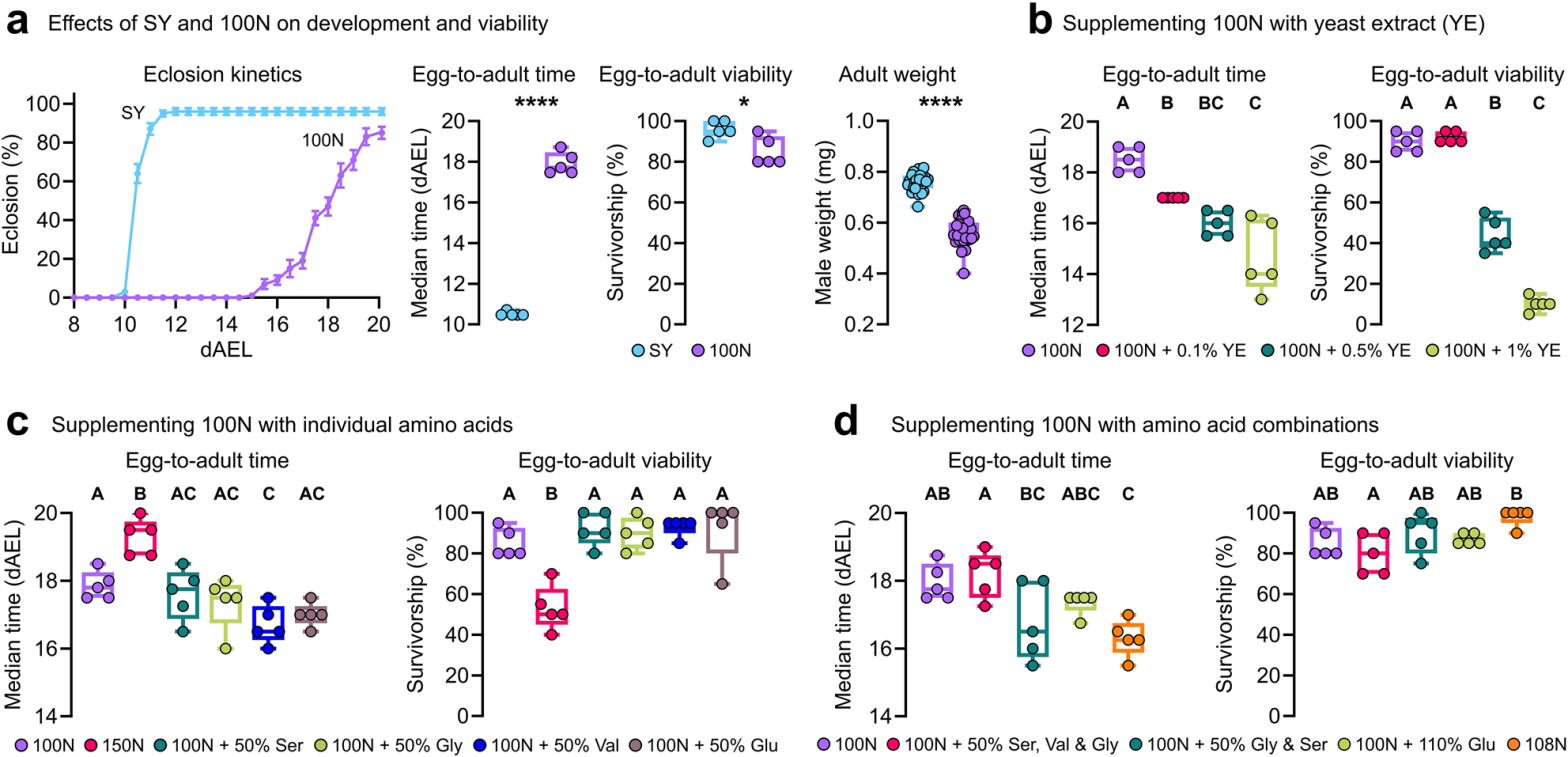
Optimizing the amino acid content of 100N for *Drosophila* pre-adulthood. (**a**), Eclosion kinetics, median egg-to-adult duration in days after-egg-lay (dAEL), mean percentage egg-to-adult survivorship, and male weight upon eclosure (mg) on SY and 100N diets. (**b**–**d**), Egg-to-adult duration (dAEL) and egg-to-adult survivorship on 100N supplemented with (**b**) 0.1%; 0.5%; or 1% of yeast extract (YE), (**c**) 50% Ser; 50% Gly; 100% Glu; 50% supplementation of all 20 aa (150N); and (**d**) 50% Ser, Val and Gly, 50% Gly and Ser, 110% Glu, and 8% supplementation of all 20 aa (108N). (**a**–**d**), five biological replicates per group of 20 individuals each. For adult male weight upon eclosure at least 30 individuals were weighted per group. (**a**), Student's unpaired *t*-test, ****p < 0.0001, *p < 0.05. **b**-**d**, One-way ANOVA followed by Tukey’s HSD test; different letters (A, B and C) represent statistically significant differences (P < 0.05).

*D. melanogaster* is unique amongst animal models as it has a fully synthetic chemically-defined (holidic) medium allowing for manipulation of individual nutrients^6^. This diet, named hereon as 100N, has been used extensively to test how nutrients impact complex adult traits such as reproduction, lifespan, behaviour, and host-microbiome interactions^6–9^. The annotation “100N” describes our standard (100) diet, which contains the minimal amount of high-quality dietary nitrogen (N) to support maximal adult fecundity and longevity^7^. However, a major limitation of 100N is that flies reared solely on it (i) are significantly delayed when compared to flies reared on sugar-yeast (SY) diet (100N: 17.95 days to eclosion; SY: 10.55 days at 25 °C), (ii) show reduced survivorship (100N: 85%; SY: 96%), and (iii) are smaller in size upon eclosion (Fig. 1a, Figure S1a), suggesting that 100N is suboptimal for growth and development. To yield a formula suitable for experiments in pre-adult stages we tested a series of supplementations involving amino acids (aa), lipids, carbohydrates, and other metabolites such as carnitine and acetic acid as suggested by metabolome profiles. Here we show that by adding 8% extra total amino acid content and doubling the amount of acetic acid in the original formula, developmental timing is reduced, egg-to-adult survivorship and fat levels in the fat body are restored, and adult body mass upon eclosure is enhanced, without compromising adult lifespan. We further demonstrate an application of the improved formula together with a *D. melanogaster* model of an inherited metabolic disorder for identifying diets of therapeutic potential.

## Results and discussion

To improve the growth and development of *D. melanogaster* reared from embryo to adulthood on the holidic diet, we made a series of tests varying individual nutrients. As a starting point, before investigating which specific nutrients could be limiting, we examined the effects of a complex mixture, water soluble yeast extract (YE). Yeast is a common component of fly media and vinegar flies are naturally attracted to it^10^. We enquired whether a component present in YE could improve the development time of flies reared on 100N. Adding 0.1% YE improved development time by 1.5-days, but while further additions progressively improved development time, they also reduced survival indicating nutritional imbalance (Fig. 1b, Figure S1b). YE is a rich source of amino acids (aa), which promote growth and development in many ways. Among these aa, Glutamate (Glu) is extensively utilized for protein synthesis and plays a critical role in central carbon and nitrogen metabolism^11^. Glycine (Gly) and serine (Ser), besides their protein synthesis function, are vital for cellular redox control and are the major source of one-carbon units, which is essential for various anabolic pathways including nucleotide synthesis^12^. Branched-chain amino acids (BCAAs), such as valine (Val), play pivotal roles in growth regulation via the activation of the growth-stimulating target of rapamycin (TOR) pathway^13,14^, as well as in muscle function and immunity^15^. Thus, we investigated whether individual supplementation of these four amino acids could confer similar benefits without compromising survival. Individually adding 50% more (in comparison to 100N) Ser, Gly, or Glu showed trends but no significant reduction in development time (Fig. 1c, Figure S1c). The only individual aa supplementation tested to produce a significant reduction in development time from egg to adult and without compromising survivorship was Val (Fig. 1c, Figure S1c). As a control we also tested the effect of supplementing 50% of all aa (150N). That, however, was highly detrimental to survival and development rate (Fig. 1c, Figure S1c). Given the positive trends observed with some of the four aa individual supplementations we next investigated whether supplementing the four in different combinations could improve development time (Fig.1d, Figure S1d). Increasing the content of both Gly and Ser by 50%, or even the content of Ser, Val and Gly by 50%, caused no significant improvement. Given Glu is an important nitrogen source, we tested whether 110% extra Glu (equal to the nitrogen content of supplementing 50% extra Ser, Val and Gly), would improve development rate, but again had no effect, suggesting the development delay is not caused by lack of nitrogen. However, as a control diet, an addition of 8% more of all 20 amino acids (108N; equal to the nitrogen content of supplementing 50% extra Ser, Val and Gly) promoted a reduction in egg-to-adult development time by 1.7-days and significantly increased survivorship (98%, Fig. 1d, Figure S1d).

**Figure 2:**
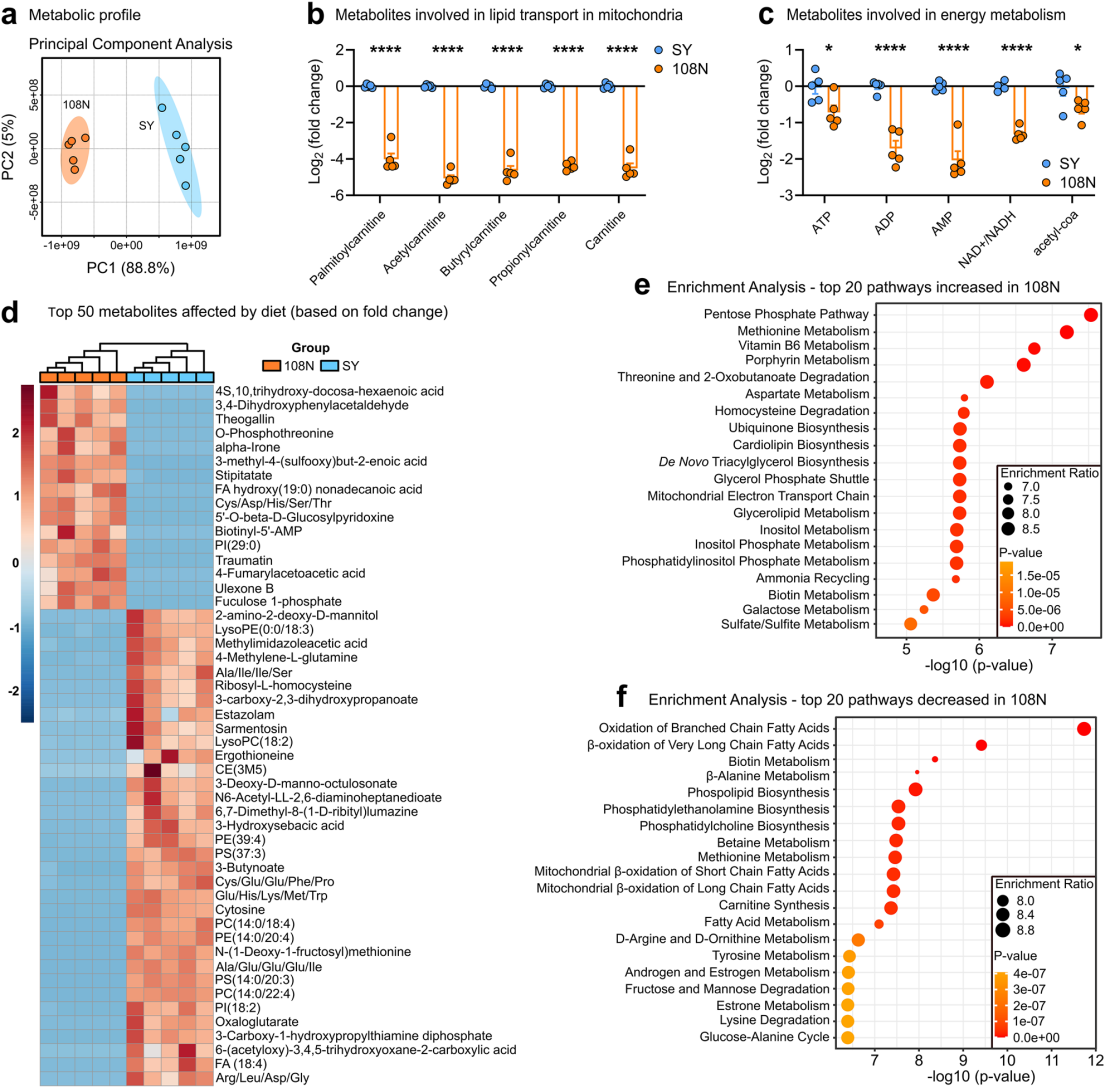
Metabolic profile comparison of 3^rd^ instar larvae reared on SY or 108N. (**a**), Principal Component Analysis (PCA) of metabolic profiles of third instar larvae reared on SY or 108N. For metabolome analysis a total of five biological replicates were performed per group, each replicate containing 10 five-day-old larvae. (**b**,**c**) Log2(fold change) of larval metabolites involved in (**b**) long-chain FA transport in mitochondria, and (**c**) energy metabolism significantly affected in 108N in comparison to SY media. Student's unpaired *t*-test, ****p < 0.0001. (**d**), Top 50 metabolites, based on fold change, significantly affected by dietary treatment (Student's unpaired *t*-test, P < 0.05). The cell color represents the z-score, *i.e.*, the standardized scores on the same scale, calculated dividing a score's deviation by the standard deviation in the row. The features are color-coded by row with red indicating high intensity and blue indicating low intensity. (**e**,**f**), Enrichment analysis showing the top 20 pathways (based on small molecule pathway database—SMPDB) (**e**) increased or (**f**) decreased in larvae reared on 108N in comparison to larvae reared on SY.

To further improve the formula, we investigated the differences between 108N- and SY-raised *Drosophila* 3^rd^ instar larvae via untargeted metabolomics (Fig. 2, Table S1,2). We identified 1065 metabolites with a high degree of confidence and found 674 that were significantly different between the two diets. In particular, lipid-, carnitine-, energy metabolism- (Fig. 2b,c), and β-oxidation-associated metabolites (Fig. 2d–f) were all lower in the 108N-raised larvae, suggesting the developmental delay may be due to an energy deficit, possibly correlated to fatty acid (FA) metabolism. Carnitines are involved in the transport of long-chain FA into mitochondria for energy production via β-oxidation^16^. We therefore attempted supplementing the 108N diet with carnitine, coconut oil (mixture rich in FA species), linolenic acid (polyunsaturated FA) and sucrose (carbon source). None of these additions improved larval performance on the 108N diet, and in many cases proved to be toxic (Figure S2a-h). These results indicate that providing energy-rich metabolites to correct the deficit suggested in the metabolic profile does not enhance the development rate. It is possible that other nutrients remain limiting and/or that fly metabolism relies on nutrients other than sugar and fats as an energy source.

**Figure 3:**
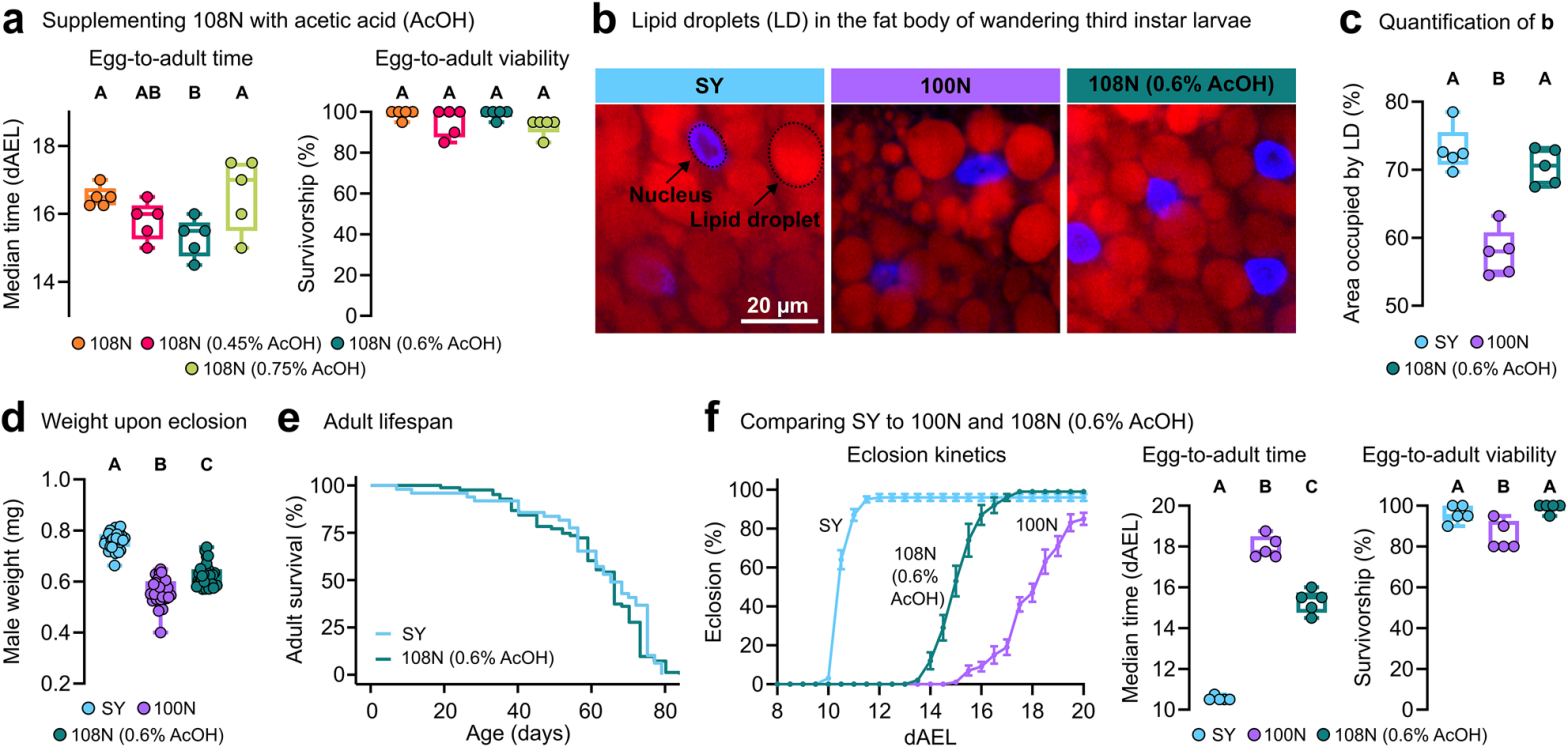
Optimizing the 108N diet for *D. melanogaster* pre-adulthood. (**a**), Egg-to-adult duration (dAEL) and egg-to-adult survivorship on 108N containing 0.45%; 0.6%; or 0.75% acetic acid (AcOH, v/v). (**b**), Lipid storage in the late-larval fat body on 100N, 108N (0.6% AcOH), or SY. Nucleic acids (blue) and lipid droplets (red), 400 × magnification. (**c**), Percentage of fat body area occupied by lipid droplets. (**d**), Adult male weight upon eclosion (mg) raised on SY, 100N, or 108N (0.6% AcOH). (**e**), Lifespan curves of adult flies fed SY food after being raised on either 108N (0.6% AcOH) or SY during their pre-adult period. (**f**), Eclosion kinetics, egg-to-adult duration (dAEL) and egg-to-adult survivorship on SY, 100N, and 108N (0.6% AcOH) diets. (**a**) and (**f**), Five biological replicates per group of 20 individuals each. (**c**), Five larvae per group (each point represents an average of 5 individual measurements per larva). (**d**), For adult male weight upon eclosure at least 30 individuals were weighed per group. (**e**), 5 to 10 replicates per group of 10 female adults each. (**a**), (**c**), (**d**), and (**f**), One-way ANOVA followed by Tukey’s HSD test; different letters (A, B and C) represent statistically significant differences (p < 0.05). i, Cox Proportional-Hazards modelling (p = 0.472). Data shown in Fig. 1a are repeated in Fig. 3d and f, to compare against 108N (0.6% AcOH).

The natural substrate of *D. melanogaster* larvae is fermenting fruit which is poor in long-chain fatty acids but rich in alcohol and acetate, a short chain FA used in the synthesis of acetyl-CoA^17,18^. We therefore tested acetic acid (AcOH) supplementation. Doubling the original content of AcOH in the holidic diet (from 0.3% v/v to 0.6% v/v) significantly reduced egg-to-adult time by 1-day compared to 108N, without compromise to survival (Fig. 3a, Figure S2i). Tissue analysis of animals reared on 108N (0.6% AcOH) showed larger adipose tissue (fat body) lipid stores in comparison to 100N-raised larvae, reaching equivalent levels to SY-raised larvae, indicating a general metabolic improvement (Fig. 3b,c). These individuals also attained significantly higher bodyweights as newly-eclosed adults, compared to 100N-raised animals (Fig. 3d), despite reaching adulthood in a shorter time. Importantly, the lifespan of adult flies that had been raised on 108N (0.6% AcOH) was no different to those raised on SY (Fig. 3e). Overall, when compared to the original holidic diet, our adjusted formula improved survivorship to 98% (equivalent to SY-raised *D. melanogaster*), reduced egg-to-adult time by 2.7 days (Fig. 3f), restored fat levels, and enhanced body mass, without compromising adult lifespan. Adult weight equivalent to SY-raised flies is not reached on 108N (0.6% AcOH), suggesting there is room for future nutritional improvements. This could involve the addition or modulation of vitamins and salts. It also remains possible that we have reached the limit of the synthetic diet environment to yield optimal fly development.

**Figure 4:**
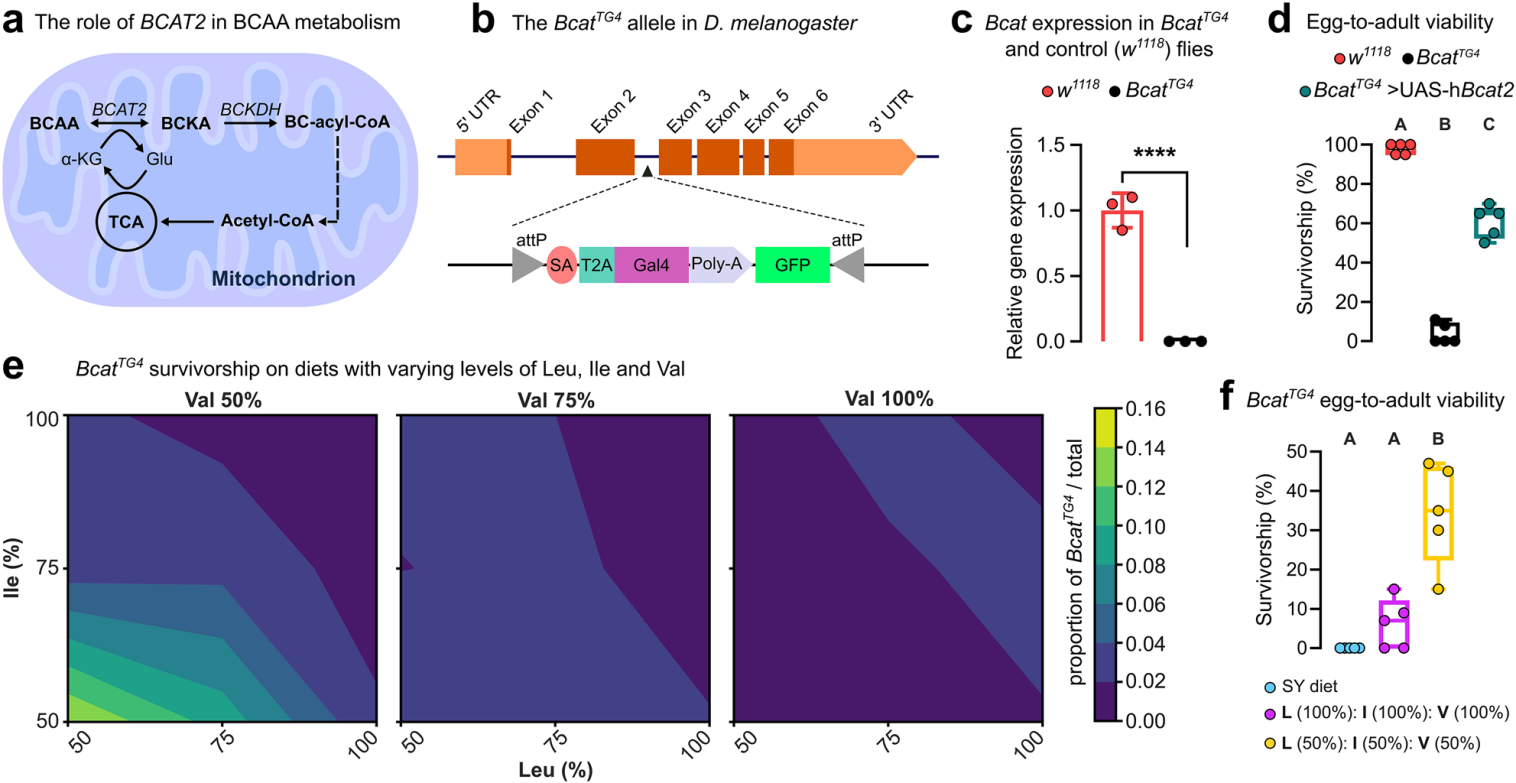
Applying the pre-adult-optimized synthetic diet in a *Drosophila* inherited metabolic disorder model. (**a**), Schematic of branched-chain amino acid (BCAA) metabolism. BCAA are converted into branched-chain α-keto acid (BCKA) by the action of BCAT2, the deficiency of which causes a rare metabolic disorder. (**b**), Schematic of Bcat^TG4^ allele in *D. melanogaster* containing the disruption T2A-GAL4 (TG4) cassette. (**c**), Validation of the *Bcat*^*TG4*^ allele, showing *Bcat* expression is not detected. *w*^*1118*^ are a wildtype control genotype. (**d**), Egg-to-adult survivorship of *Bcat*^*TG4*^ flies and *Bcat*^*TG4*^ expressing the human orthologue *Bcat2*, when reared on 108N (0.6% AcOH). (**e**), *Bcat*^*TG4*^ survivorship contour plots resulting from a 3-way nutrient (Leu, Ile, Val) dose response array (27 diets total, 8–9 replicates performed per diet). (**f**), Egg-to-adult survivorship of *Bcat*^*TG4*^ flies raised on SY, 108N (0.6% AcOH), and 108N (0.6% AcOH) with 50% BCAA content. (**d**) and (**f**), five biological replicates per group of 20 individuals each. (**c**), Student's unpaired *t*-test, ****p < 0.0001. (**d**) and (**f**), One-way ANOVA followed by Tukey’s HSD test. Different letters (A, B and C) represent statistically significant differences (p < 0.001).

A synthetic diet on which *D. melanogaster* can be raised is particularly valuable in the modelling of human metabolic disorders, where phenotypic abnormalities often manifest prior to adulthood^19^. For example, human disorders such as Phenylketonuria manifest with rapid neurological decline prior to adulthood mitigated by a custom diet with single aa restriction that drastically improves health outcomes^20^. However, the majority of metabolic disorders lack therapeutic diets^21^. To demonstrate the potential of our diet as a tool to find new dietary treatments, we applied it in a model of human Branched Chain Amino Acid (BCAA) Transaminase (*BCAT2*) deficiency, a rare disorder associated with developmental delay, intellectual disability, seizures, and movement defects^21^. *BCAT2* catalyzes the first step in Valine (Val), Leucine (Leu) and Isoleucine (Ile) catabolism^22^ and patients have high plasma and tissue levels of these aa^21^ (Fig. 4a).

We used a *D. melanogaster Bcat* allele (*Bcat*^TG4^) that comprises a T2A-GAL4 cassette inserted within the second intron of *Bcat*, which abolishes expression of the full-length transcript and truncates protein translation^23^ (Fig. 4b,c). Egg-to-adult survival for *Bcat*^*TG4*^ homozygotes on 108N (0.6% AcOH) is 4% and this was rescued to 60% via expression of a human ortholog *BCAT2* transgene suggesting fly and human genes are functionally equivalent (Fig. 4d). We next tested *Bcat*^*TG4*^ flies and their survival relative to sibling controls in a nutrigenomic array of 27 BCAA-modified diets where each BCAA (Leu, Ile, and Val) was co-varied across three concentrations (50%, 75% and 100%). *Bcat*^*TG4*^ flies had highest relative survival when reared on a diet where all three BCAAs were reduced by 50%, while no benefit was observed from restricting one or two BCAA at a time in any combination (Fig. 4e, Figure S3). Reducing Val levels to 50% whilst keeping Leu and Ile at 100% had a drastic negative impact in median egg-to-adult duration, suggesting that maintaining a BCAA balance appears important for development (Figure S3). Tracking *Bcat*^*TG4*^ flies developing in isolation on the 50% all BCAA diet (to determine absolute survival, rather than relative to genetic controls) revealed a striking improvement in survivorship (from ~ 5% to 34%, Fig. 4f). These findings mirror diet responses observed in BCAT2 deficiency patients^21^ and observations from a mouse BCAT2 model^24^. This highlights the potential of our modified synthetic diet for nutrigenomic studies of fly disease models for which effective diet formulations are lacking. More broadly, our diet expands the study of nutrient-gene interactions to the entire life cycle of *Drosophila,* thus permitting the exploration of even complex processes such as growth and development.

## Materials and methods

### Fly stocks and base diet

The synthetic (holidic) diet optimizing experiments were performed on *w*^1118^ (Bloomington stock #3605). For experiments involving *Bcat* mutant flies, *Bcat*^*TG4*^ mutants (Bloomington stock #79238) were first outcrossed to *w*^1118^ for removal of y^1^ and then maintained over fluorescently marked balancer chromosome (FM7a, ChFP)^25^. Rescue with human ortholog was performed by cross with UAS-h*BCAT2* (Bloomington stock #84835). *Bcat*^*TG4*^/ FM7a, ChFP females were crossed to FM7a, ChFP/Y; UAS-hBCAT2/UAS-hBCAT2 males. First instar larvae without ChFP maker were selected, corresponding to hemizygous males *Bcat*^*TG4*^/Y; UAS-hBCAT2/ + . The viability of hemizygous *Bcat*^*TG4*^ males containing one copy of UAS-h*BCAT2* was then compared to that of hemizygous *Bcat*^*TG4*^ males without the UAS-h*BCAT2* transgene. Dietary manipulations were conducted using the synthetic diet^6,7^ (Table S3). The sugar-yeast (SY) diet was prepared as per standard media (Table S4). All stocks of *Drosophila melanogaster* were maintained at 18 °C on sugar-yeast medium under natural photoperiod conditions. For experiments, flies were reared in incubators at 25 °C and 45% humidity in specific diets as described below.

### Testing holidic diet improvements

The modifications tested with the holidic diet include manipulation of amino acid levels, supplementation with yeast extract (Bacto™ Yeast Extract, Thermo Fisher Scientific), sucrose (Sigma-Aldrich, S1888), organic virgin coconut oil (Macro Wholefoods Market), Linolenic acid (L2376-500MG, Merck), L-Carnitine hydrochloride (Merck, C0283-5G), and acetic acid (Fisher, A/0400/PB15). The solutions of yeast extract, sucrose, acetic acid, and carnitine were prepared in Milli-Q water. Appropriate volumes of these solutions, with the exception of Linolenic acid and coconut oil, were added to the solid food surface and left for 24 h in a 25 °C incubator to allow for complete diffusion. 300 µL was added to each vial as we found that this is the maximum volume of liquid that would be fully absorbed and diffuse into the solidified holidic diet within a 24 h period and allowed for concentrations where amino acids remained soluble. For Linolenic acid and coconut oil, appropriate volumes were added to the holidic diet prior to setting and homogenized with help of a magnetic stirrer before dispensing into vials. Diets with varied amino acid concentrations were prepared by modifying the base holidic diet recipe (Table S3).

### Survival and developmental timing

Embryos were collected from population cages containing approximately 200 females and 50 males (5 to 10 days old) laying for 6 h on apple juice agar plates supplemented with yeast paste. Embryos were then washed with distilled water and transferred to a new plate not containing yeast paste and left for 24 h to hatch. Twenty first instar larvae were transferred to each vial for the different diets. For each diet, at least five replicate vials were tested (n = 100 individuals). Vials were monitored every 12 h for a period of 20 days and the numbers of pupae and adults were recorded. For creating the pupariation kinetics or eclosion kinetics curves, data were visualized as the cumulative percentage of individuals at each life stage as a function of time (days) from egg lay. Box plots depicting each biological replicate data point were also generated to represent the median time of development (in days) and mean survival (in percentage) for egg-to-pupa and egg-to adult stages. Significant differences in median time of development and mean survival were analyzed using multiple comparison one-way ANOVA followed by Tukey’s HSD test, where each group was compared against each other group within an experiment (GraphPad Prism 9). Groups with different letters (“A”, “B”, etc.) indicate they are significantly different from one another, while groups with the same letter are indistinguishable (p-values are shown in Figure legends).

### Metabolic profiling

Five replicates of 10 five-day-old larvae from each genotype and diet condition were collected, washed in PBS, blotted dy, and weighed. Samples were then transferred to 1.5 mL safe lock microtubes tubes (Eppendorf), flash frozen in liquid nitrogen, and stored at -80 °C. Thawed larvae were homogenized using a disposable pestle in 20 µL of ice-cold extraction solvent consisting of 2:6:1 chloroform:methanol:water with 2 µM of (CHAPS, CAPS, PIPES and TRIS) acting as internal standards. Once ground additional solvent was added to a final ratio of 20 µL of extraction solvent per mg of larvae. Samples were vortexed for 30 s and then sonicated in an ice-water bath for 10 min and centrifuged at 4 °C (22,000 × g for 10 min). The supernatant was then transferred to a glass vial for LC–MS based metabolomic analysis. 20 µL of each extract were combined to make a pooled quality control (PQC) sample.

LC–MS was performed using a Dionex Ultimate 3000 UHPLC coupled to an QExactive Plus mass spectrometer (Thermo Scientific). Samples were analyzed by hydrophilic interaction liquid chromatography (HILIC) following a previously published method^26^. The chromatography utilized a ZIC-pHILIC column 5 µm, 150 × 4.6 mm with a 20 × 2.1 mm ZIC-pHILIC guard column (both Merck Millipore, Australia) (25 °C). A gradient elution of 20 mM ammonium carbonate (A) and acetonitrile (B) (linear gradient time-%B: 0 min-80%, 15 min-50%, 18 min-5%, 21 min-5%, 24 min-80%, 32 min-80%) was utilized. Flow rate was maintained at 300 μL/min. Samples were stored in the autosampler (6 °C) and 10 μL was injected for analysis. MS was performed at 70,000 resolution operating in rapid switching positive (4 kV) and negative (− 3.5 kV) mode electrospray ionization (capillary temperature 300 °C; sheath gas flow rate 50; auxiliary gas flow rate 20; sweep gas 0; probe temp 120 °C). Samples were randomized and processed in a single batch with intermittent analysis of PQC samples to ensure reproducibility. For accurate metabolite identification, a standard library of ~ 500 metabolites was analyzed before sample testing and accurate retention time for each standard was recorded. This standard library also forms the basis of a retention time prediction model used to provide putative identification of metabolites not contained within the standard library^27^.

### Metabolic profiling data analysis

Acquired LC–MS/MS data was processed in an untargeted fashion using open source software IDEOM, which initially used msConvert (ProteoWizard)^28^ to convert raw LC–MS files to mzXML format and XCMS to pick peaks to convert to .peakML files^29^. Mzmatch was subsequently used for sample alignment and filtering^30^. Metabolites were identified based on accurate mass (< 2 ppm) and comparison of their retention time against that determined for compounds in the standard library or predicted on the basis of their physiochemical characteristics^27^. Only metabolites that were identified with a level of confidence equal to or greater than 6 in IDEOM were used for downstream functional and statistical analyses, using MetaboAnalyst 5.0^31^. Global metabolic variations due to genotype and diet were visualized using PCA. One-way ANOVA [false discovery rates (FDR) < 0.05] was used to identify significant changes in metabolite levels. A heatmap was created to visualize the 50 topmost affected metabolites (based on fold change).

### Assessment of lipid environment by confocal microscopy

The effects of SY, 100N and 108N supplemented with 0.6% AcOH on the lipid storage of wandering third instar larvae were assessed using fluorescence microscopy. Fat bodies were dissected in PBS (Ambion) and subjected to lipid staining with Nile Red N3013 Technical grade (Sigma-Aldrich) and nucleic acid staining with DAPI (Sigma-Aldrich). Tissues were fixed in 4% PFA (Electron Microscopy Science) for 20 min and stained with 0.5 µg/mL Nile Red and 10 µg/mL DAPI in PBS + 0.1% Triton X-100 for 15 min. After staining, samples were washed in PBS and slide were mounted in Vectashield (Vector Laboratories). Microscopies were performed in CV1000 Disc Confocal Microscope (Olympus Life Science) at 400 × magnification. Images were analyzed using ImageJ software. The percentage of area occupied by lipid droplets per fat body samples was averaged from three independent measurements of randomly chosen sections of 2500 µm^2^. A total of five biological replicates were assessed for each dietary condition, each replicate consisting of a single tissue from a single larva. Results were analyzed using One-way ANOVA followed by Tukey’s HSD test (GraphPad Prism 9).

### Male weight upon eclosure

The adult weight upon eclosure of male flies reared on SY, 100N, or 108N + 0.6% AcOH was assessed using a SE2 Ultramicrobalance (Sartorius, Goettingen, Germany). At least 30 flies were weighed upon eclosure. Results were analyzed using One-way ANOVA followed by Tukey’s HSD test (GraphPad Prism 9).

### Adult lifespan

The survival of flies reared from egg-to-adult on 108N (0.6% AcOH) was compared to those reared on the SY diet. Upon eclosion, female adults from either diet were transferred onto SY diet and survivorship was checked every other day, at the same time when flies were transferred to fresh media. Five to ten replicates per group of ten flies each were performed. Fly survival was recorded using the software DLife^32^ and Cox Proportional-Hazards modelling was used for analysis (R, package survival). Difference in survival between groups was analysed using a linear model (base R, “lm”) and post-hoc comparisons from the emmeans package.

### BCAA-modified diet array

Large numbers of developmentally synchronized embryos obtained from multiple cages containing *Bcat*^*TG4*^/FM7a, ChFP flies were collected and washed in distilled water, and transferred to 15 mL tubes containing PBS. Once the embryos had settled (1 min), excess PBS was removed. Using a micropipette set to 5 µL and a tip cut to widen the bore, small volumes of embryos were aspirated and then dispensed into vials onto the medium surface. This was performed for each of the 27 modified synthetic diets tested with varied levels (100%, 75% and 50%) of leucine, isoleucine and valine replicated at least 8 times (Table S4). Vials were checked daily for 30 days and the total number of adult flies were counted with the survival proportion of *Bcat*^*TG4*^ (*Bcat*^*TG4*^/ total flies) calculated. The linear mixed model (R package: lme4) was fitted to analyse the effects of diet, genotype, and their interaction on mean *Bcat*^*TG4*^ survival proportion (number of *Bcat*^*TG4*^ flies out of total), with replicates treated as a random effect.

### Real time PCR

The expression of *Bcat* was measured in *Bcat*^*TG4*^ homozygous flies and *w*^*1118*^ control flies. A total of five whole flies were used for a single biological replicate and three replicates were taken from each genotype. RNA isolation with TriSure (Bioline) was performed following the manufacturer’s instructions. RNA purity and concentration were evaluated by spectrophotometry (NanoDrop® ND-1000, NanoDrop Technologies). RNA samples were then diluted to 1 µg/µL and stored at − 80 °C. cDNA was generated using the Superscript III reverse transcriptase (NEB). RT-qPCR analysis was performed in a 7500 Real Time PCR System (Applied Biosystems) using the SYBR® Green Reagent protocol (Applied Biosystems) and the following pairs of primers: *Bcat* Forward 5’ CCGCCTATTCACCGATCACA3’; *Bcat* Reverse 5’ TACGCCTTCATGCCCTCAAA3’; *RpL11* 5’ CGATCCCTCCATCGGTATCT3’; *RpL11* Reverse 5’ AACCACTTCATGGCATCCTC3’. Relative expression was calculated using the 2^(-∆∆Ct) method and *RpL11* expression as a housekeeping gene. The results were analyzed by the Shapiro–Wilk normality test (p > 0.05) to test for normality, before comparing the transcript levels between the two treatment groups using two-tailed Student's t-tests (p < 0.05 (GraphPad Prism 9).

### Supplementary Information

Supplementary Figure S1.

Supplementary Figure S2.

Supplementary Figure S3.

Supplementary TableS1.

Supplementary TableS2.

Supplementary TableS3.

Supplementary TableS4.

Supplementary TableS5.

Supplementary Legends.

## Data Availability

All data generated or analysed during this study are included in this published article and its supplementary information files except for the raw metabolomics dataset which can be found at the MetaboLights repository upon publication, www.ebi.ac.uk/metabolights/MTBLS9556.
