# Supplementary figures and images for: A defined diet for pre-adult Drosophila melanogaster
Source: Sci Rep. 2024 Mar 23;14:6974. doi: 10.1038/s41598-024-57681-z (PMC10960813; doi:10.1038/s41598-024-57681-z)

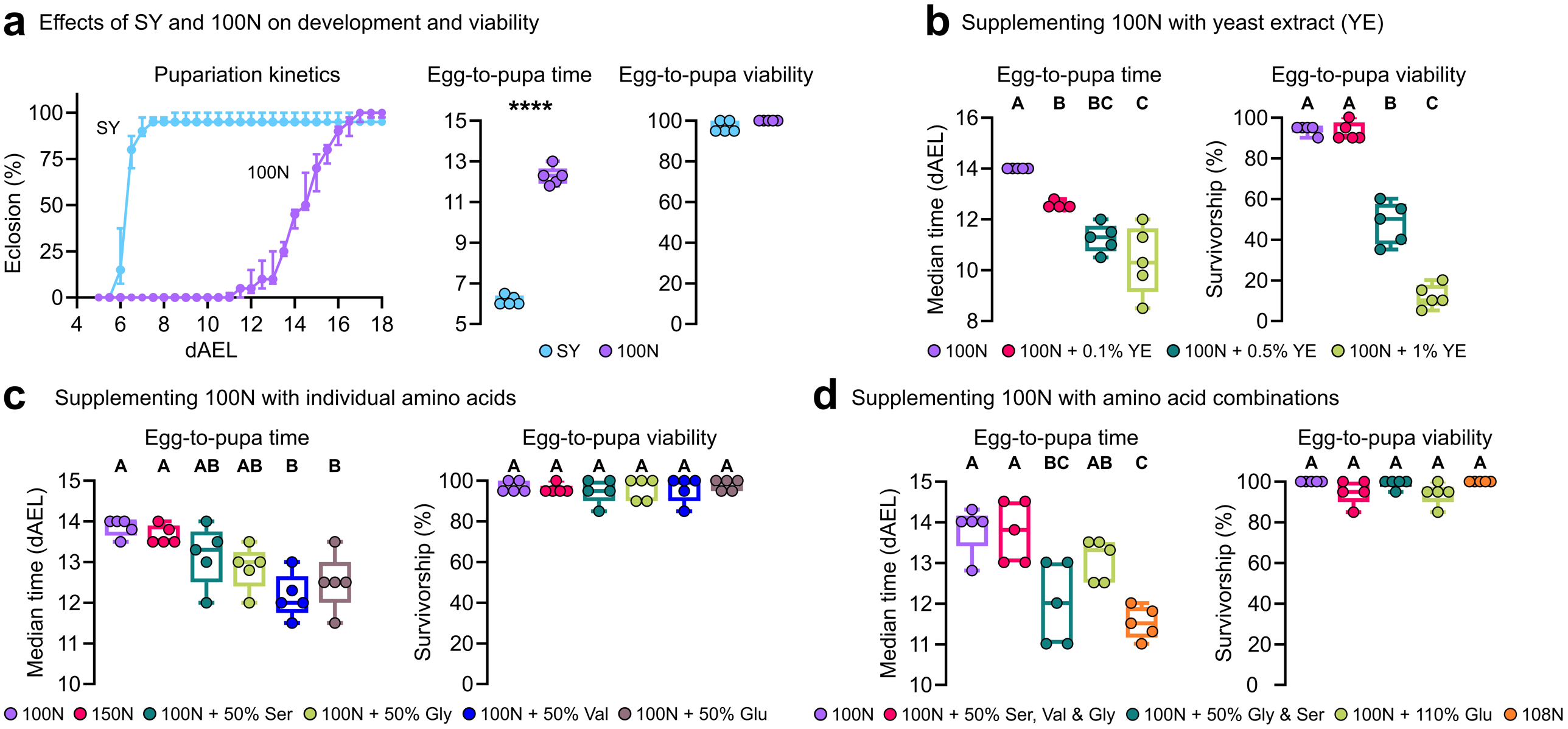

Supplement: Supplementary file 1 — Supplementary Figure S1. [file 41598_2024_57681_MOESM1_ESM.jpg]
