# Supplementary material for: A defined diet for pre-adult Drosophila melanogaster
Source: Sci Rep. 2024 Mar 23;14:6974. doi: 10.1038/s41598-024-57681-z (PMC10960813; doi:10.1038/s41598-024-57681-z)
Supplement: Supplementary file 9 — Supplementary Legends. [file 41598_2024_57681_MOESM9_ESM.docx]

**Supplementary figure legends**

**Figure S1. Optimizing the amino acid content of 100N for *D. melanogaster* pre-adulthood. a,** Pupariation kinetics, median egg-to-pupa duration in days after-egg-lay (dAEL), and mean percentage egg-to-pupa survivorship on SY and 100N diets. **b-d,** Egg-to-pupa duration (dAEL) and egg-to-pupa survivorship on 100N supplemented with (**b**) 0.1%; 0.5%; or 1% of yeast extract (YE), (**c**) 50% supplementation of all 20 aa (150N); 50% Ser; 50% Gly; 50% Val; or 50% Glu, and (**d**) 50% Ser, Val and Gly; 50% Gly and Ser; 110% Glu; or 8% supplementation of all 20 aa (108N). **a**-**d**, five biological replicates per group of 20 individuals each. **a**, Student's unpaired *t*-test, ****p < 0.0001. **b-d**, One-way ANOVA followed by Tukey’s HSD test; different letters (A, B and C) represent different letters (a and b) represent statistically significant differences (P < 0.05).

**Figure S2. Testing the effect of non-nitrogen based nutrient supplementations on 108N diet. a, c, e, g,** and **i,** Median egg-to-pupa duration in days after-egg-lay (dAEL) and mean percentage egg-to-pupa survivorship of flies reared on 108N supplemented with (**a**) 1 mg/L; 10 mg/L; or 100 mg/L carnitine (C), (**c**) 0.05%; 0.1%; or 0.5% coconut oil (CO), (**e**) 0.3 g/L; 0.1 g/L; or 0.03 g/L linolenic acid (LA), (**g**) 10%; 25%; or 50% sucrose (S), and (**i**) 0.45%; 0.6%; or 0.75% acetic acid (AcOH). **b, d, f,** and **h,** Median egg-to-adult duration in days after-egg-lay (dAEL) and mean percentage egg-to-adult survivorship of flies reared on 108N supplemented with (**b**) 1 mg/L; 10 mg/L; or 100 mg/L carnitine (C), (**d**) 0.05%; 0.1%; or 0.5% coconut oil (CO), (**f**) 0.3 g/L; 0.1 g/L; or 0.03 g/L linolenic acid (LA), and (**h**) 10%; 25%; or 50% sucrose (S). One-way ANOVA followed by Tukey’s HSD test; different letters represent statistically significant differences (p < 0.05).

**Figure S3. Survival and median egg-to-adult time in response to varying BCAA levels.** Total number of flies and median egg-to-adult duration (dAEL) for each one of the 27 diets resulting from a 3-way nutrient (Leu, Ile, Val) dose response array, plus a diet with 0% content of BCAA. Eight to nine replicates performed per diet. One-way ANOVA followed by Tukey’s HSD test; different letters represent statistically significant differences (p < 0.05).

**Supplementary excel file titles**

**Table S1. Normalised metabolome dataset for the two dietary conditions.**

**Table S2. Enrichment analysis output for the metabolomics dataset.**

**Table S3. Nutrigenomics phenotyping raw data.**

**Table S4. Diet recipes and calculators.**

**Table S5. Raw data for all plots (dev time/viability, adult weight, fat body).**
